# Rhynchophylline Ameliorates Endothelial Dysfunction via Src-PI3K/Akt-eNOS Cascade in the Cultured Intrarenal Arteries of Spontaneous Hypertensive Rats

**DOI:** 10.3389/fphys.2017.00928

**Published:** 2017-11-15

**Authors:** Hui-Feng Hao, Li-Mei Liu, Chun-Shui Pan, Chuan-She Wang, Yuan-Sheng Gao, Jing-Yu Fan, Jing-Yan Han

**Affiliations:** ^1^Tasly Microcirculation Research Center, Peking University Health Science Center, Beijing, China; ^2^Department of Integration of Chinese and Western Medicine, School of Basic Medical Sciences, Peking University, Beijing, China; ^3^Department of Physiology and Pathophysiology, Peking University Health Science Center, Beijing, China; ^4^Key Laboratory of Molecular Cardiovascular Science, Ministry of Education, Beijing, China; ^5^Key Laboratory of Microcirculation, State Administration of Traditional Chinese Medicine of the People's Republic of China, Beijing, China; ^6^Key Laboratory of Stasis and Phlegm, State Administration of Traditional Chinese Medicine of the People's Republic of China, Beijing, China; ^7^State Key Laboratory of Core Technology in Innovative Chinese Medicine, Beijing, China

**Keywords:** Src kinase, PI3K/Akt, endothelial function, renal artery, hypertension

## Abstract

**Objectives:** To examine the protective effect of Rhynchophylline (Rhy) on vascular endothelial function in spontaneous hypertensive rats (SHRs) and the underlying mechanism.

**Methods:** Intrarenal arteries of SHRs and Wistar rats were suspended in myograph for force measurement. Expression and phosphorylation of endothelial nitric oxide (NO) synthase (eNOS), Akt, and Src kinase (Src) were examined by Western blotting. NO production was assayed by ELISA.

**Results:** Rhy time- and concentration-dependently improved endothelium-dependent relaxation in the renal arteries from SHRs, but had no effect on endothelium-independent relaxation in SHR renal arteries. Wortmannin (an inhibitor of phosphatidylinositol 3-kinase) or PP2 (an inhibitor of Src) inhibited the improvement of relaxation in response to acetylcholine by 12 h-incubation with 300 μM Rhy. Western blot analysis revealed that Rhy elevated phosphorylations of eNOS, Akt, and Src in SHR renal arteries. Moreover, wortmannin reversed the increased phosphorylations of Akt and eNOS induced by Rhy, but did not affect the phosphorylation of Src. Furthermore, the enhanced phosphorylations of eNOS, Akt, and Src were blunted by PP2. Importantly, Rhy increased NO production and this effect was blocked by inhibition of Src or PI3K/Akt.

**Conclusion:** The present study provides evidences for the first time that Rhy ameliorates endothelial dysfunction in SHRs through the activation of Src-PI3K/Akt-eNOS signaling pathway.

## Introduction

Hypertension, a major risk factor leading to the development of cardiovascular diseases, is associated with functional and structural alterations in both conduit and resistance arteries (Salvetti et al., 2014; Liu et al., 2015; Bartoloni et al., 2017). Substantial evidences have shown that endothelial dysfunction characterized by down-regulated activity of endothelial nitric oxide synthase (eNOS) is critically involved in vascular dysfunction in hypertension (Panza et al., 1993; Vanhoutte, 1996; Higashi et al., 2012; Nguyen et al., 2013; Yannoutsos et al., 2014). Further, the severity of endothelial dysfunction correlates with the threatening outcome of hypertension and predicts future cardiovascular events (Perticone et al., 2001; Tang and Vanhoutte, 2010). Improving endothelial dysfunction has beneficial effects for ameliorating the hazards related to hypertension (Modena et al., 2002; Zhou et al., 2004; Koh et al., 2007). The endothelium-dependent vasodilation which is mainly determined by the activity of eNOS, is commonly used for evaluating endothelial function of the vascular and has been proved to be depressed in the renal arteries from the spontaneous hypertensive rats (SHRs) (Liu et al., 2012; Wang et al., 2014).

*Uncaria rhynchophylla (UR)*, also named “Gou-Teng” in China, is a traditional Chinese medical herb that has been used to treat ailments of the cardiovascular and central nervous systems (Zhang et al., 2004; Chou et al., 2009; Zhou and Zhou, 2010). Rhynchophylline (Rhy), a pharmacologically active substance isolated from *UR*, is widely used for the treatment of hypertension (Sutter and Wang, 1993; Zhou and Zhou, 2010; Ndagijimana et al., 2013). Antihypertensive action of Rhy is observed in a number of animal models (Zhang et al., 2010; Zhou and Zhou, 2010), and has been attributed to its possible vasodilatory action via inhibiting L-type Ca^2+^ channel and/or decreasing calcium sensitivity in the smooth muscle cells (Zhang et al., 2004; Li et al., 2013; Hao et al., 2014). However, it remains unclear whether Rhy could ameliorate endothelial dysfunction in hypertension.

The present study investigated the effect of Rhy on endothelium-dependent relaxation of the renal arteries from SHRs, and explored the underlying mechanism. Our results revealed a protective effect of Rhy on endothelial dysfunction in hypertensive rats and suggested that activation of Src-PI3K/Akt-eNOS signaling may mediate this action of Rhy.

## Materials and methods

### Reagents

Sodium nitroprusside (SNP), acetylcholine (ACh), phenylephrine (PE), wortmannin, PP2, nitro-L-arginine (NLA) and indomethacin were purchased from Sigma (St. Louis, MO, USA). Rhy was obtained from Fengshanjian Company (Kunming, China). Indomethacin (10^−5^ M) was prepared with equimolar Na_2_CO_3_ in distilled water. Rhy was dissolved in hydrochloric acid (0.1 M) to a concentration of 0.1 M, and diluted in distilled water for further usage (Hao et al., 2014). SNP, ACh, and PE were dissolved in distilled water. Wortmannin and PP2 were prepared in dimethyl sulphoxide (DMSO).

### Animals

Male SHRs and Wistar rats, 8–10 months old, were supplied by Vital River Laboratories (Beijing, China). All animal care and experimental procedures in this investigation were approved by Animal Experimentation Ethics Committee of Peking University Health Science Center and complied with the Guide for the Care and Use of Laboratory Animals published by the US National Institute of Health (NIH Publication, 8th Edition, 2011).

### Tissue preparations

Rats were sacrificed by CO_2_ suffocation and the second branches of renal interlobar arteries were dissected out and cut into ring segments, ~2 mm in length, in ice-cold Krebs-Ringer bicarbonate solution (118.3 mM NaCl, 4.7 mM KCl, 2.5 mM CaCl_2_, 1.2 mM MgSO_4_, 1.2 mM KH_2_PO_4_, 25 mM NaHCO_3_, and 11.1 mM glucose) (Gao et al., 2007). To determine the effect of prolonged treatment of Rhy on the endothelium-dependent vasodilation, the arterial rings were incubated in Dulbecco's Modified Eagle's Media (DMEM, Gibco, Grand Island, NY, USA) with 10% fetal bovine serum (FBS, Gibco), 100 IU penicillin and 100 μg/mL streptomycin for 1, 4, 8, 12 h at 37°C with vehicle or Rhy (300 μM). In another set of experiments, SHR renal arteries were treated with Rhy (30, 100, and 300 μM) for 12 h at 37°C to assess the concentration dependency. In still another set of experiments, the arteries were incubated for 12 h with vehicle or Rhy (300 μM) at 37°C in the presence or absence of wortmannin (an inhibitor of phosphatidylinositol 3-kinase, 10 μM) or PP2 (an inhibitor of Src, 10 μM) (Anselm et al., 2007).

### Isometric vessel tension studies

Isometric vessel tension was determined as previously described (Liu et al., 2014). Briefly, the arteries were suspended using two stainless tungsten wires in the chamber of a Multi Myograph (620M, Danish, Myo Technology A/S, Aarhus, Denmark) with 5 mL Krebs solution maintained at 37°C and constantly bubbled with 95% O_2_-5% CO_2_. The arteries were firstly brought to their optimal tension (~2.5 mN) and maintained for 90 min for equilibration. Then the arteries were contracted with 60 mM KCl. Following several washes in warmed Krebs solution, the relaxations in response to ACh (0.003–10 μM) were examined in the arteries pre-contracted with PE (3 μM). Thereafter, the endothelium-independent vasodilation to SNP (0.003–10 μM) was examined after 30 min-incubation with NLA (10^−4^ M) and indomethacin (10^−5^ M).

### Western blot analysis

The arteries cultured for 12 h as mentioned above were homogenized in RIPA lysis buffer (1 μg/mL leupeptin, 5 μg/mL aprotinin, 100 μg/mL PMSF, 1 mM sodium orthovanadate, 1 mM EDTA, 1 mM EGTA, 1 mM sodium fluoride, and 2 μg/mL β-glycerolphosphate). The homogenate was sonicated (5 s for 3 times, 4°C) and centrifuged (20,000 g, 20 min, 4°C). The supernatant was collected, and proteins were quantified and solubilized in 5× loading buffer. After separated on SDS-PAGE, proteins were electro-transferred to a polyvinylidene difluoride (PVDF) membrane (Millipore, Bedford, MA). Non-specific binding of antibody was blocked by incubation with 5% nonfat dry milk in Tris-buffered saline with 0.1% Tween 20 (TBST) for 1 h at room temperature. The PVDF membrane was then subjected to two brief washes with TBST and incubated in TBST containing the primary antibodies of appropriate dilution for overnight at 4°C. After two more washes in TBST, the PVDF membrane was incubated for 1 h with horseradish-peroxidase conjugated secondary antibodies at room temperature. After three washes in TBST, the blots were visualized with an enhanced chemiluminescence reagent kit (Applygen Technologies Inc, Beijing, China). Quantitation of the proteins was performed using Quantity One 4.6.2 software (Bio-Rad, Hercules, California, USA). Primary antibodies used for Western blotting were as follows: rabbit anti-phospho-eNOS (1:500; CST, Boston, USA), rabbit anti-eNOS (1:500; CST, Boston, USA), rabbit anti-phospho-Akt (1:1000; CST, Boston, USA), rabbit anti-Akt (1:1000; CST, Boston, USA), rabbit anti-phospho-Src (1:1000; CST, Boston, USA), rabbit anti-Src (1:2000; CST, Boston, USA), and mouse anti-β-actin (1:10000; Calbiochem, San Diego, CA, USA).

### Nitric oxide (NO) assessment

Tissues for NO detection was prepared as described for Western blot analysis. The content of NO was determined using NO assay kit (S0021, Beyotime Institute of Biotechnology, Beijing, China), according to the instruction of the manufacturer. Briefly, Griess Reagent I and II in the kit were rewarmed at room temperature before use. The standard NaNO_2_ was prepared in different concentrations (0, 1, 2, 5, 10, 20, 60, 100 μM), and added to a 96-well plate along with samples (50 μL/well). Thereafter, Griess Reagent I and II were added into the wells (50 μL of each reagent/well), mixed thoroughly, and the absorbance of each well was determined at 540 nm in wavelength.

### Statistical analysis

Data were expressed as means ± SEM. When mean values of two groups were compared, Student's *t*-test for unpaired observations was used. Mean values of more than two groups were compared using one-way ANOVA test, with the Student-Newman-Keuls test for *post hoc* testing of multiple comparisons. In vessel tension studies, individual points were compared using two-way ANOVA with Bonferroni's post-tests. In each experiment, “n” represented the number of rats used. *p* < 0.05 is considered statistically significant.

## Results

### Endothelial function is impaired in SHR renal arteries

**Figure 1:**
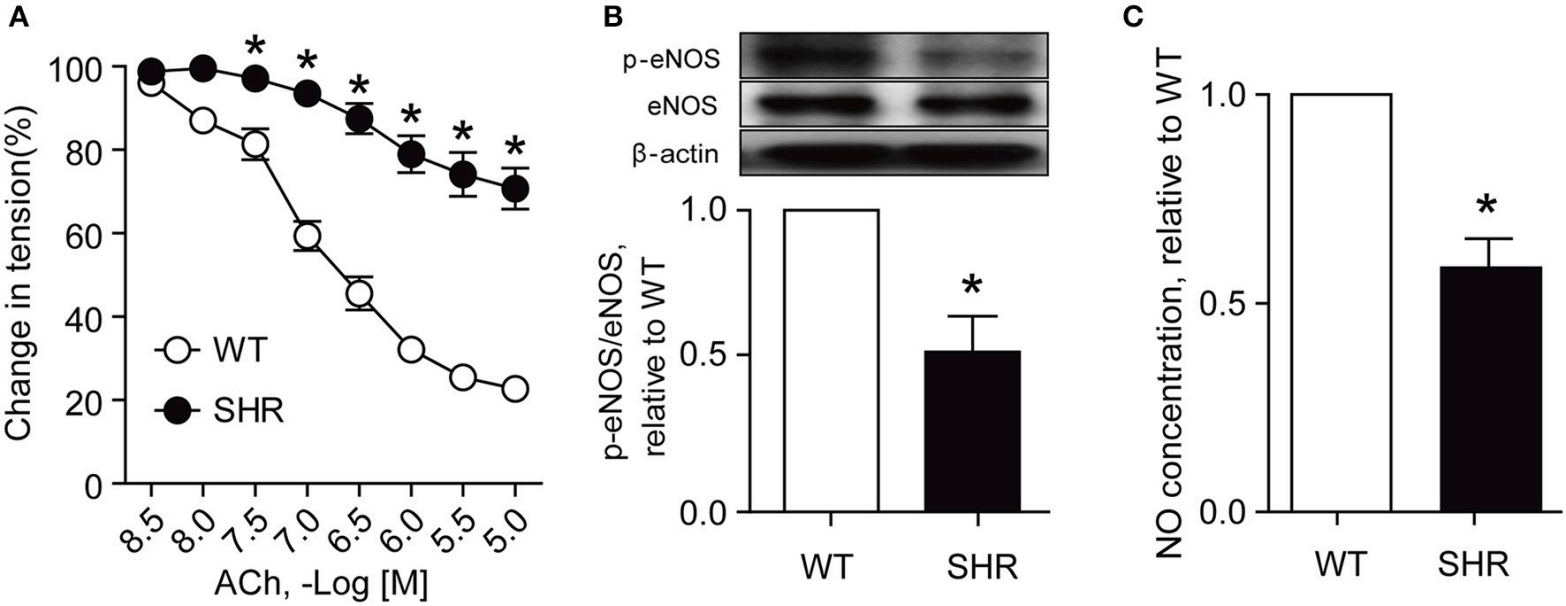
Endothelial function is impaired in SHR renal arteries. Endothelium-dependent relaxation (**A**, *n* = 6), eNOS phosphorylation (**B**, *n* = 4) and nitric oxide (NO) production (**C**, *n* = 4) in renal arteries from Wistar rats (WT) and SHRs. ^*^p < 0.05 vs. WT.

Acetylcholine (ACh) is a vasodilator which relaxes vessels mainly by increasing production of NO by activation of eNOS (Huang et al., 1995). The phosphorylation of eNOS plays a major role in NO production and endothelium-dependent vasodilation (Kobayashi et al., 2003). The present study showed that as compared with those from Wistar rats, renal arteries from SHRs exhibited a less sensitive response to ACh in endothelium-dependent relaxation (Figure 1A), a decreased expression of eNOS phosphorylation (Figure 1B), and a reduced NO production (Figure 1C), suggesting that the endothelial function in renal arteries of SHRs is impaired.

### Rhy ameliorates endothelial dysfunction in the renal arteries from SHRs

**Figure 2:**
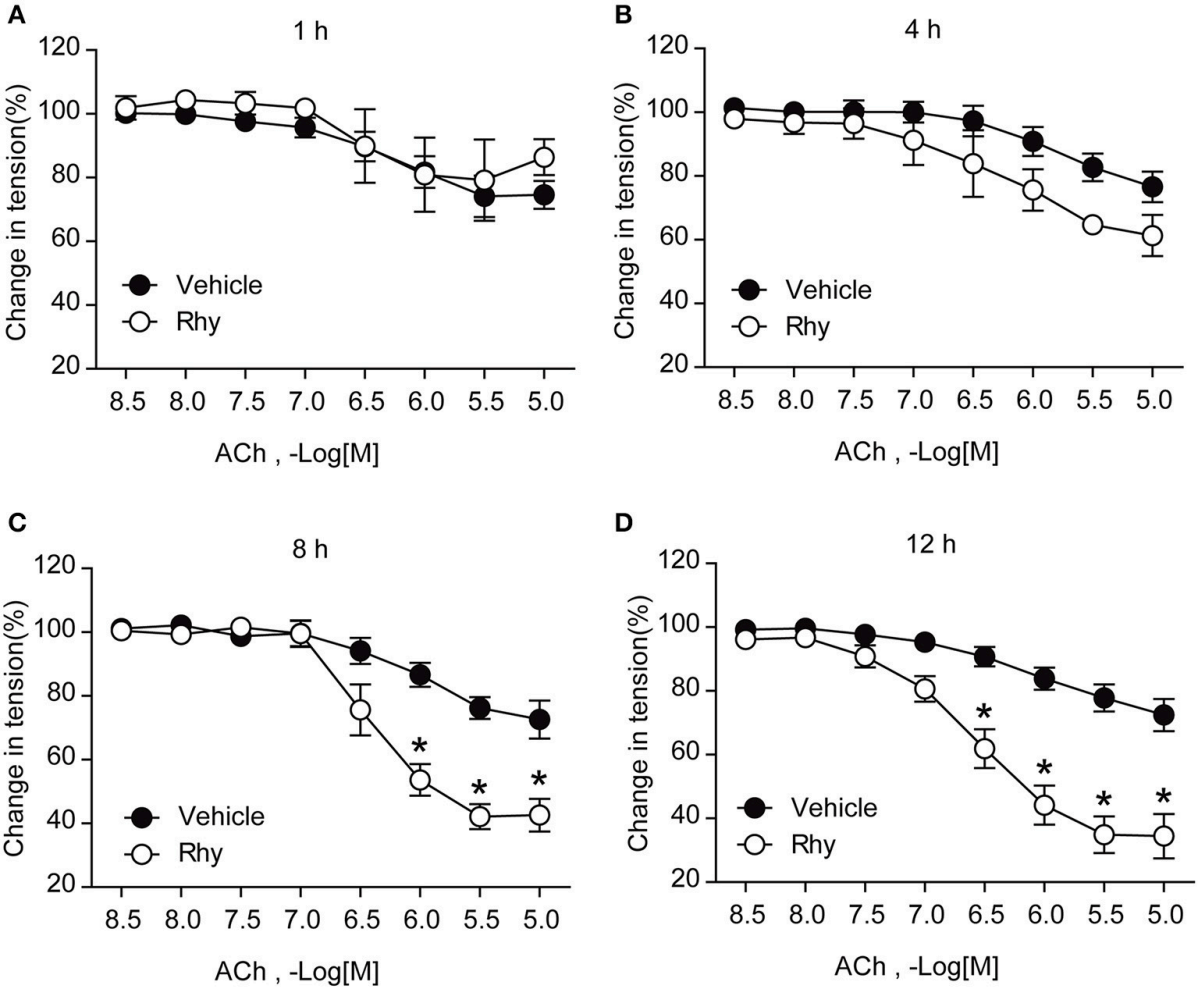
Rhy improves endothelial function of SHRs in a time-dependent manner. Endothelium-dependent relaxations in response to ACh in SHR renal arteries incubated with Rhy (300 μM) for 1 h **(A)**, 4 h **(B)**, 8 h **(C)**, and 12 h **(D)**. *n* = 4; ^*^p < 0.05 vs. vehicle.

**Figure 3:**
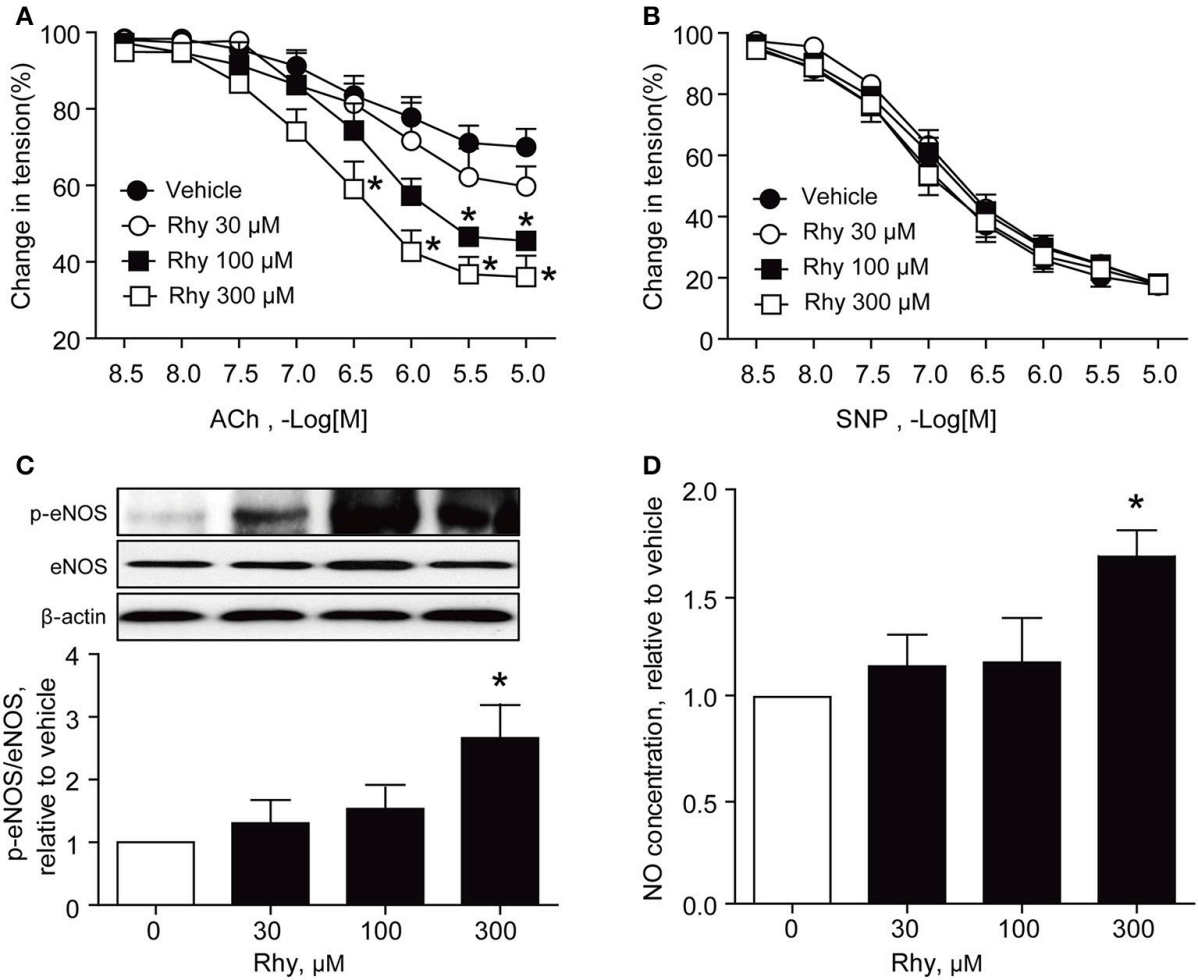
Rhy restores endothelial function concentration-dependently in SHR renal arteries. **(A)** Endothelium-dependent relaxation in response to ACh in the arteries from SHRs incubated with Rhy (30, 100, or 300 μM) or vehicle for 12 h. **(B)** Endothelium-independent relaxation response to SNP in SHR renal arteries. **(C)** Effects of Rhy on eNOS phosphorylation in the renal arteries from SHRs. **(D)** Effects of Rhy on NO production in the renal arteries from SHRs. *n* = 6; ^*^p < 0.05 vs. vehicle.

The endothelium-dependent relaxation of renal arteries from SHRs was further tested as to its response to ACh in the presence of Rhy. The results showed that Rhy improved the endothelium-dependent relaxation in response to ACh in SHR renal arteries in a time-dependent manner (Figure 2), as well as a concentration-dependent manner (Figure 3A). By contrast, no effect of Rhy was observed on endothelium-independent relaxations in response to sodium nitroprusside (SNP) at all concentration tested (Figure 3B). Noticeably, Rhy incubation did not significantly change the contraction multitude caused by PE (Supplemental Figure 1). While, Rhy enhanced eNOS phosphorylation (Figure 3C) and NO production (Figure 3D) in the renal arteries from SHRs, highlighting eNOS as its target.

### Rhy improves endothelial function in SHRs but not that in wistar

**Figure 4:**
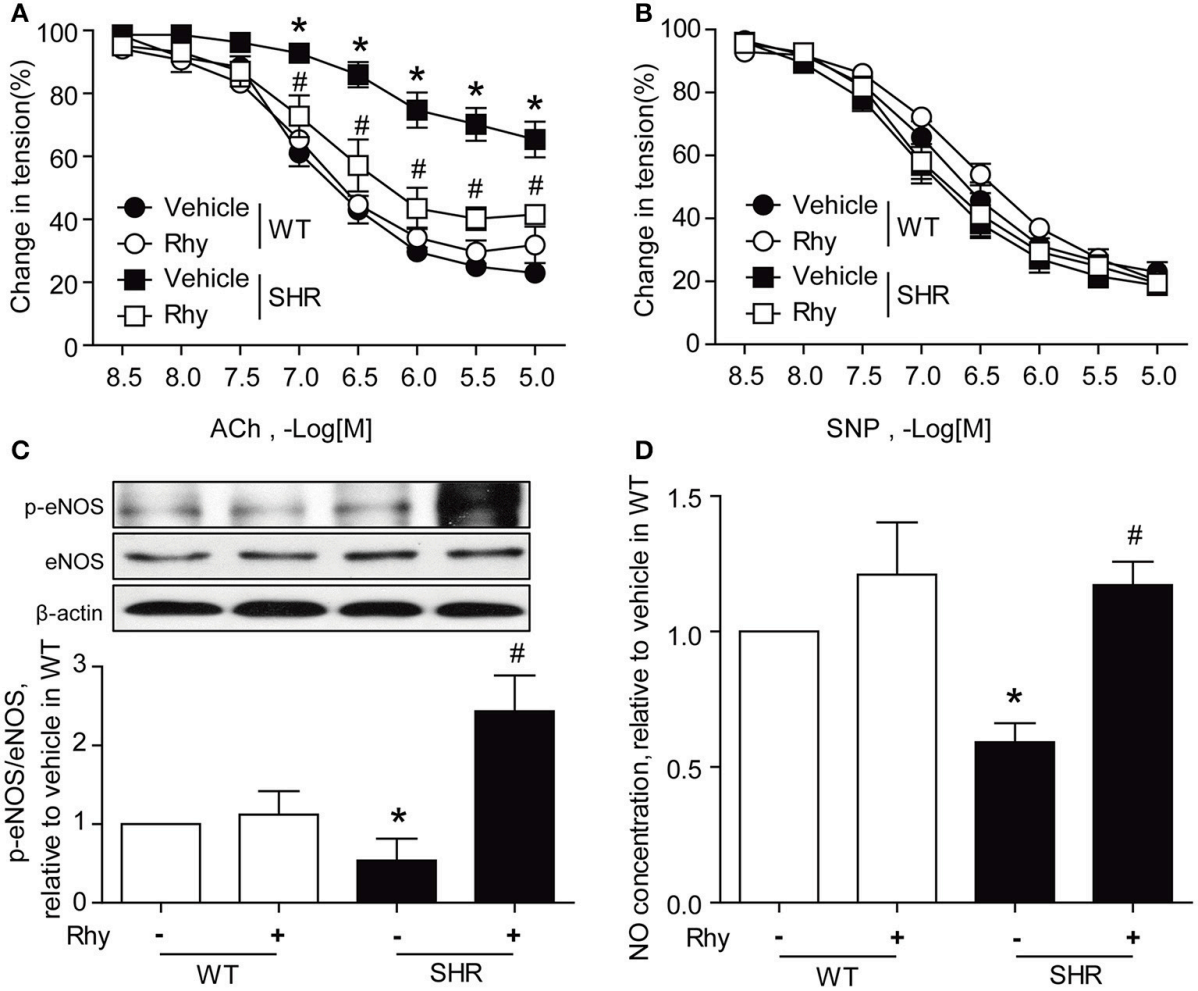
Rhy ameliorates endothelial dysfunction in SHR renal arteries. **(A)** Effects of 12 h-incubation with Rhy (300 μM) on endothelium-dependent relaxation in response to ACh in the renal arteries from Wistar and SHRs. **(B)** Effects of 12 h-incubation with Rhy (300 μM) on endothelium-independent relaxation in response to SNP in the renal arteries from Wistar and SHRs. **(C)** Effects of Rhy (12 h, 300 μM) on eNOS phosphorylation in the rat renal arteries. **(D)** Effects of Rhy (12 h, 300 μM) on NO production in the rat renal arteries. *n* = 6 **(A,B)**, *n* = 4 **(C,D)**; ^*^p < 0.05 vs. vehicle in WT; #*p* < 0.05 vs. vehicle in SHRs.

Exposure to Rhy (300 μM) for 12 h markedly augmented ACh-induced endothelium-dependent relaxation in the renal arteries from SHRs, resulting in a response curve similar to that in Wistar rats (Figure 4A). The endothelium-dependent relaxation of the renal arteries from Wistar rats responded to ACh stimulation more sensitively than that of SHRs, and this response was not affected by the presence of Rhy (Figure 4A). The endothelium-independent relaxation in response to SNP was assessed for SHR and Wistar rats in both the presence and absence of Rhy, and no difference was observed among groups (Figure 4B). Exposure to Rhy (300 μM) for 12 h increased eNOS phosphorylation (Figure 4C) and NO production (Figure 4D) in SHR renal arteries, but did not affect eNOS phosphorylation (Figure 4C) or NO level (Figure 4D) in renal arteries of Wistar rats.

### Rhy restores endothelial function through Src-PI3K/Akt-eNOS cascade in SHR renal arteries

**Figure 5:**
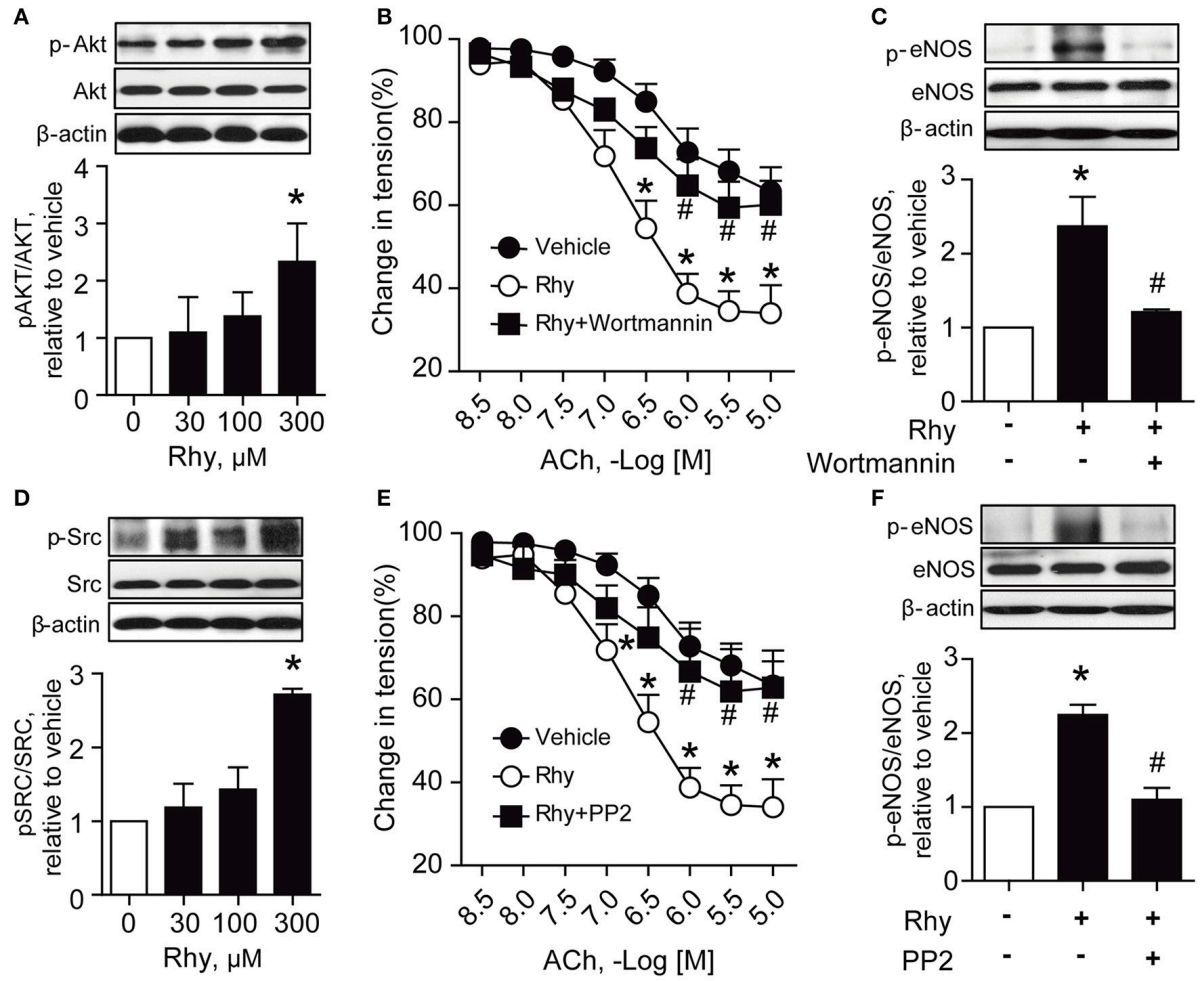
Endothelial function improved by Rhy is Src- and Akt-dependent in SHR renal arteries. **(A)** Effects of 12 h-incubation with Rhy (30, 100, or 300 μM) or vehicle on Akt phosphorylation in the arteries from SHRs (*n* = 4). **(B)** Effects of wortmannin (a PI3K inhibitor, 10 μM) on the Rhy-improved relaxation (*n* = 6). **(C)** Effects of wortmannin (a PI3K inhibitor, 10 μM) on the Rhy-increased eNOS phosphorylation in SHR renal arteries (*n* = 4). **(D)** Effects of incubation with Rhy (30, 100, or 300 μM) or vehicle for 12 h on Src phosphorylation in the arteries from SHRs (*n* = 4). **(E)** Effects of PP2 (a Src inhibitor, 10 μM) on the Rhy-improved endothelium-dependent relaxation in SHR renal arteries (*n* = 6). **(F)** Effects of PP2 (a Src inhibitor, 10 μM) on the Rhy-increased eNOS phosphorylation in SHR renal arteries (*n* = 4). ^*^p < 0.05 vs. vehicle; #*p* < 0.05 vs. Rhy.

**Figure 6:**
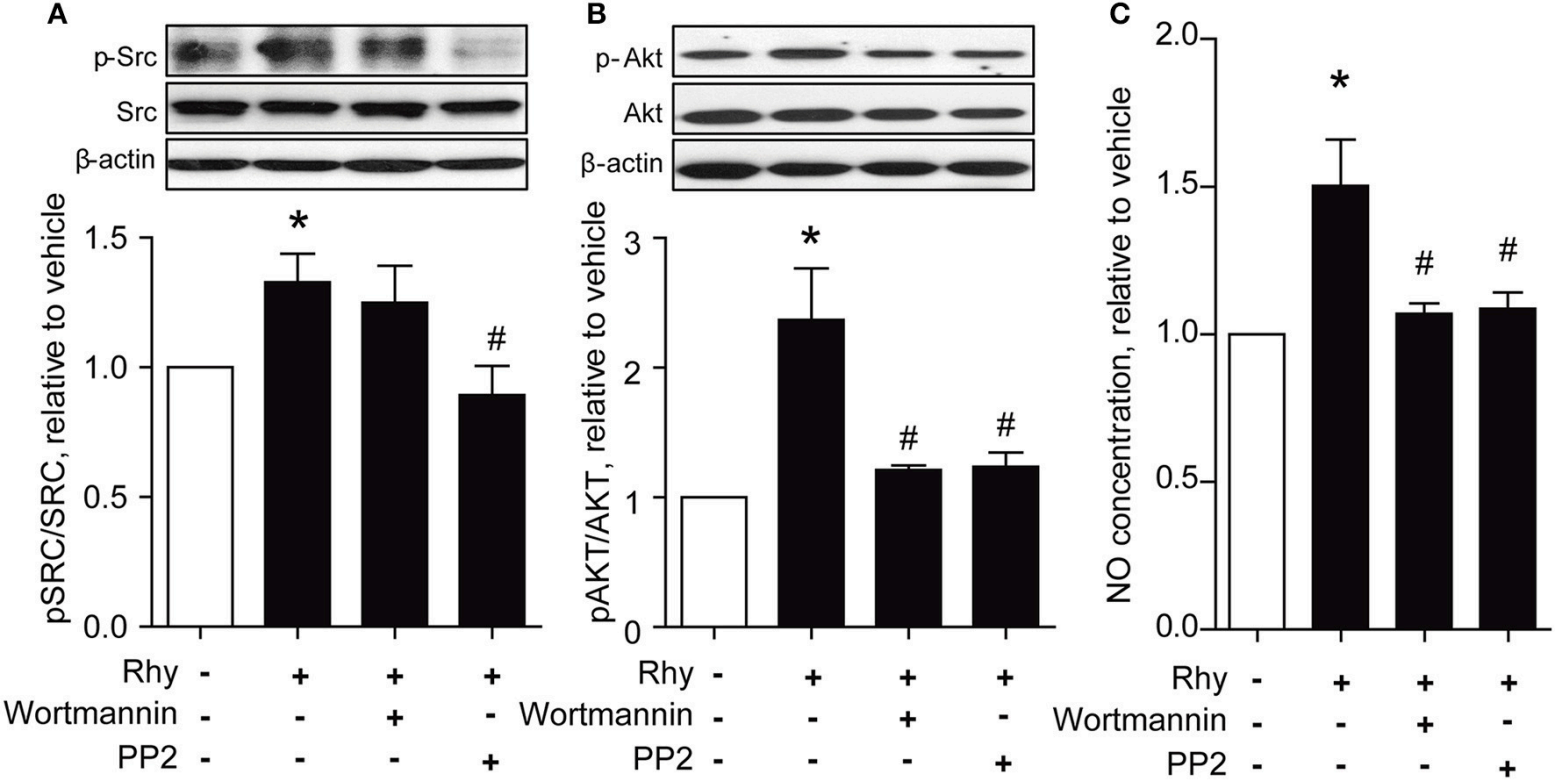
Rhy improves endothelial function via Src/Akt pathway in the renal arteries from SHRs. **(A)** Effects of wortmannin (10 μM) and PP2 (10 μM) on the Rhy (300 μM, 12 h)-enhanced phosphorylations of Src in SHR renal arteries. **(B)** Effects of wortmannin (10 μM) and PP2 (10 μM) on the Rhy (300 μM, 12 h)-enhanced phosphorylations of Akt in SHR renal arteries. **(C)** Effects of wortmannin (10 μM) and PP2 (10 μM) on the Rhy (300 μM, 12 h)-elevated NO production in SHR renal arteries. *n* = 4; ^*^p < 0.05 vs. vehicle; #*p* < 0.05 vs. Rhy.

PI3K/Akt signaling is recognized as a stimulator of eNOS (Anselm et al., 2007; Sampaio et al., 2007). Thus, we explored the effect of Rhy on phosphorylation of Akt in SHR renal arteries, and found a significantly increased Akt phosphorylation in SHR renal arteries after incubation with 300 μM of Rhy for 12 h (Figure 5A). The involvement of Akt in the effect of Rhy was demonstrated by the finding that the Rhy-improved endothelium-dependent relaxation (Figure 5B) and increased eNOS phosphorylation (Figure 5C) were reversed by addition of wortmannin (an inhibitor of PI3K, 10 μM). Src is known as a regulator of PI3K/Akt-eNOS signaling (Haynes et al., 2003). We then assessed the effect of Rhy on Src phosphorylation in the arteries from SHRs and found that Src phosphorylation was significantly increased by Rhy under conditions similar to that for Akt (Figure 5D). The role of Rhy in endothelium-dependent relaxation (Figure 5E) and eNOS phosphorylation (Figure 5F) was abolished by PP2 (a Src inhibitor, 10 μM). As expected, the Rhy-increased Src phosphorylation was blunted by PP2 but not but by wortmannin (Figure 6A), while, the Rhy-enhanced Akt phosphorylation was reversed by PP2 and wortmannin (Figure 6B). In addition, both PP2 and wortmannin inhibited the increase of NO production stimulated by Rhy (Figure 6C). Taken together, Rhy improved endothelial function through Src-PI3K/Akt-eNOS signaling pathway in SHR renal arteries.

## Discussion

The present study provides *in vitro* evidences that Rhy ameliorates endothelial dysfunction in SHR renal arteries via stimulating eNOS phosphorylation and elevating NO production, and thus restores endothelium-dependent relaxation. Further evidences indicate that activating Src-PI3K/Akt-eNOS signaling pathway is involved in the beneficial effects of Rhy observed. These findings suggest that Rhy might be valuable for the treatment of vascular dysfunction associated with hypertension.

SHRs are widely used as an animal model to study the pathophysiology and management of hypertension, and a range of abnormalities have been reported in SHRs relative to the normal counterparts, including defection in both Akt-dependent and Akt-independent signaling pathways (Iaccarino et al., 2004; Hsiao et al., 2008), an increased vasoactive intestinal peptide-mRNA expression (Avidor et al., 1989), and a reduced content of vasopressin in the brain (Lang et al., 1981), among others, which are supposed to be either a causal or a compensatory factor for the hypertension on SHRs. As to the L-arginine/NO pathway in SHRs, the data varies, which probably depends on the age of the animals. It was reported that renal and vascular nitric oxide synthase in young SHRs were upregulated (Vaziri et al., 1998), while substantial other reports have shown that the endothelium-dependent relaxation was significantly repressed in elder SHRs (Liu et al., 2012; Wang et al., 2014). The present study demonstrated that SHRs which were 8–10 months old, exhibited impaired endothelium-dependent relaxation in the renal arteries compared to Wistar rats which expressed similar vasoreactions to Wistar-Kyoto rats (WKYs) (Liu et al., 2012), indicating that these arteries are suitable for studying the role of Rhy in endothelium-dependent relaxation.

Rhy has reportedly multiple pharmacological activities, which benefit cardiovascular and central nervous system diseases, including hypertension, bradycardia, arrhythmia, sedation, vascular dementia, epileptic seizures, drug addiction, and cerebral ischemia (Zhou and Zhou, 2010). The study on the vasodilatory potential of Rhy has received increasing attention in view of its hypotensive effect. It's well-documented that Rhy exerted vasodilation effect via blocking calcium signaling in the smooth muscle cells (Zhang et al., 2004; Li et al., 2013; Hao et al., 2014), however, the protective action of Rhy in endothelial cells has not been reported. Kuramochi and colleagues reported that a crude aqueous extract of *U. rhynchophylla (Miq.)*, which contains a number of indole alkaloids including Rhy, induces both endothelium-independent relaxation and endothelium-dependent relaxation in the isolated rat aorta depending on the concentrations of extract applied (Kuramochi et al., 1994). However, the results regarding the effect of purified Rhy revealed no endothelium-dependent relaxation effect for this reagent (Zhang et al., 2004). The present study demonstrated that Rhy had an endothelium-dependent relaxing effect in arteries isolated from SHRs, wherein an impaired eNOS activity occurred. However, this effect was not observed in the arteries from Wistar rats in the present study, a result in line with that of Zhang et al. (2004), implying that the effect of Rhy is to restore the impaired eNOS activity but not to increase the activity of normally functioning eNOS. As abnormal eNOS occurs commonly among patients with hypertension (Cengiz et al., 2015; ALrefai et al., 2016), these findings highlight that the patients with endothelial dysfunction might benefit from the endothelium-protective potential of Rhy.

It's reported that the vascular Akt activity, charactered by phosphorylations at Ser^473^, is downregulated in a number of hypertensive rat models, including in SHRs (Inoue et al., 2009; Lima et al., 2009; Xing et al., 2013). Importantly, phosphorylation of Akt at Ser^473^ contributes to the activation of eNOS (Dimmeler et al., 1999; Haynes et al., 2000; Du et al., 2001; Yamamoto et al., 2007). Src regulates the activity of PI3K/Akt signaling, and contributes to improving endothelial dysfunction (Haynes et al., 2003; Matsui et al., 2007; Su et al., 2009; Nemoto et al., 2011). The present study showed that Rhy upregulated the phosphorylation of Akt and Src, and specific inhibition of Akt or Src both abolished the attenuating effect of Rhy on the impaired eNOS. Further, the findings that inhibiting Src abrogated the increase of Akt phosphorylation caused by Rhy, and that inhibition of PI3K/Akt failed to influence the phosphorylation of Src, suggested that PI3K/Akt is regulated by Src in the underlying signaling. Taken together, these results revealed Src-PI3K/Akt-eNOS cascade as a fundamental signaling mediating the protective action of Rhy on endothelial function. Src also activates ERK and MEK, in addition to Akt. Whether other MEKs are affected by Rhy, if yes, whether these signaling pathways also contribute to the vascular effect of Rhy is a question worthy to be further investigated.

In summary, the present study demonstrated that Rhy was capable of activating Src-PI3K/Akt-eNOS cascade and improving endothelium-dependent relaxation in the renal arteries from SHRs. These results provided new insight for better understanding the pharmacology of Rhy, supporting the clinical use of Rhy in patients with hypertension.

## Author contributions

H-FH performed most of the experiments, analyzed the data, and wrote the manuscript. L-ML conceived and supervised the study, helped in conducting the experiments, wrote the manuscript, and provided funding. C-SP and C-SW assisted in preparing experimental materials, collecting, and analyzing data. Y-SG advised for study design, and provided funding. J-YF wrote the manuscript. J-YH conceived and supervised the study, and provided funding.

### Conflict of interest statement

The authors declare that the research was conducted in the absence of any commercial or financial relationships that could be construed as a potential conflict of interest.
