# Supplementary material for: Rhynchophylline Ameliorates Endothelial Dysfunction via Src-PI3K/Akt-eNOS Cascade in the Cultured Intrarenal Arteries of Spontaneous Hypertensive Rats
Source: Front Physiol. 2017 Nov 15;8:928. doi: 10.3389/fphys.2017.00928 (PMC5694770; doi:10.3389/fphys.2017.00928)
Supplement: Supplementary file 1 [file DataSheet1.DOC]

**Supplemental Figure and Figure legend**


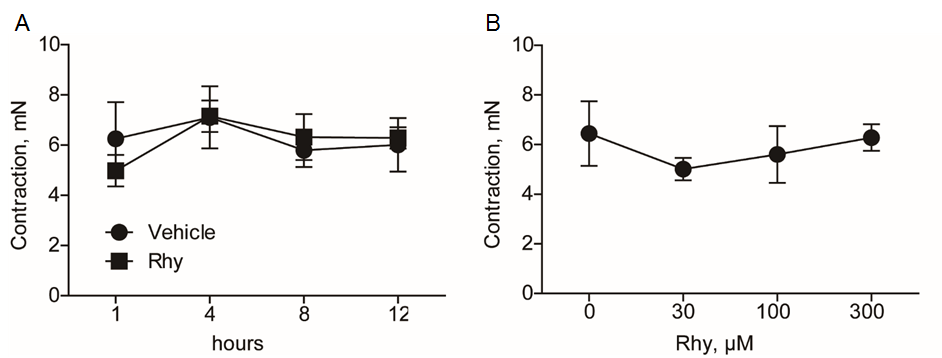


**Supplemental Figure 1. Rhy incubation did not affect the contraction induced by phenephrine (PE).** (**A**) Effects of incubation with vehicle or Rhy (300 µM) for 1, 4, 8 or 12 hours on the vasoconstriction to PE; n=4. (**B**) Effects of incubation of Rhy (0, 30, 100 or 300 µM) for 12 hours on the vasoconstriction to PE; n=6.
